# 2344. Clinical Profile and Immunologic Response from COVID-19 Vaccine Among People Living with HIV Enrolled in a Treatment Hub in the Philippines

**DOI:** 10.1093/ofid/ofad500.1966

**Published:** 2023-11-27

**Authors:** Mark John Festin, Regina Berba, Christine P Ramos, Jodor Lim

**Affiliations:** University of the Philippines-Philippine General Hospital, Caloocan, National Capital Region, Philippines; University of the Philippines Manila, Manila, National Capital Region, Philippines; University of the Philippines - Philippine General Hospital, Manila, National Capital Region, Philippines; University of the Philippines Manila, Manila, National Capital Region, Philippines

## Abstract

**Background:**

SARS-CoV-2 has caused more than 1 million deaths worldwide. Several studies showed that People with HIV had higher rates of hospitalization and mortality with COVID-19 compared with people without HIV but data on serologic response to COVID-19 vaccine among People Living with HIV (PLHIV) is still limited.

**Methods:**

A prospective cohort study on determination of serologic response to COVID-19 vaccine among completely vaccinated PLHIV enrolled in a treatment hub in the Philippines until December 31, 2021 was done. Baseline demographics were collected via chart review. History of COVID-19 infection was also asked during enrollment, on follow-up and at the end of 6 months after the 2^nd^ dose of vaccination.

Blood Extraction were done on enrollment and on follow-up visit to the treatment hub. All the specimen were processed using the SARS-CoV2 ( RocheElecsys Anti-SARS-CoV2 spike (S) protein assays.

**Results:**

A total of 261 PLHIV who received vaccination from February 11, 2021 to December 29, 2021 were enrolled in the study. The participants received any of the following vaccine brands: CoronoVac/Sinovac (41%), Oxford-AstraZeneca (20.7%), Pfizer-BioNTech (21.5%), Moderna (14.2% and Janseen (2.7%). Univariate analysis showed that presence of opportunistic infection, HIV Viral load , Antiretroviral(ARV) Status, COVID-19 Vaccine brand were all significant predictors of antibody response. On multiple regression analysis, only the Vaccine Brand and Viral load remained significant. Sinovac gave a lower median antibody level and seroconversion rate as compared to other vaccine brands. HIV Viral load > 1000 copies/ml was predictive of lower antibody response.

**Figure:**
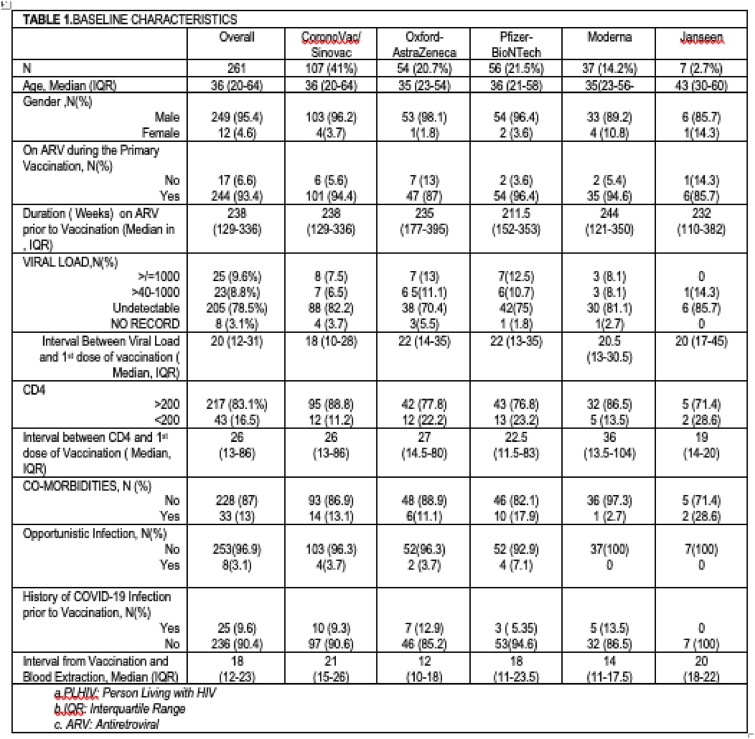

**Figure:**
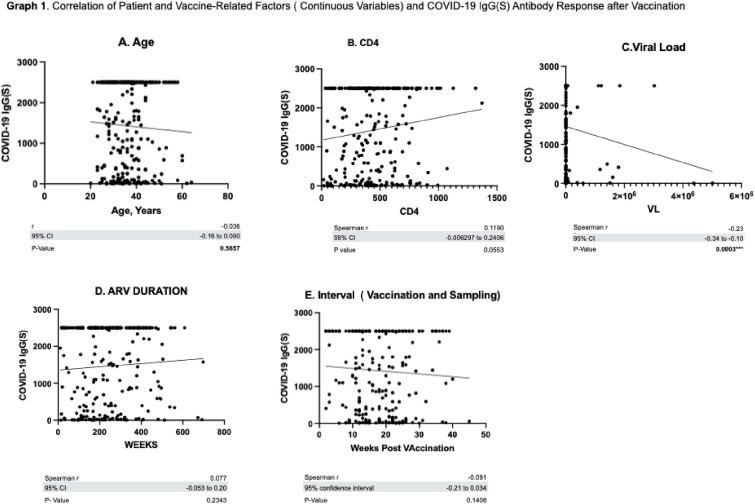

**Figure:**
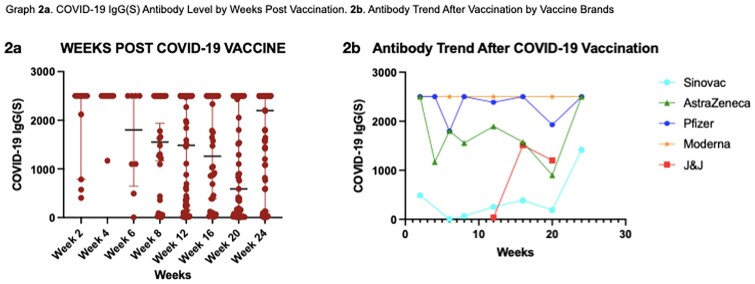

**Conclusion:**

In this study among PLHIV, the type of vaccine and HIV viral load were significant predictors of antibody response to COVID-19 Vaccine. Specifically, Sinovac COVID-19 Vaccine gave a lower Median SARS-CoV-2 IgG(S) Antibody and Seroconversion rate as compared to other Vaccine brands. HIV Viral load > 1000 copies/ml was predictive of lower antibody response to COVID-19 Vaccine . Overall, the antibody response peaked at 2-4 weeks after vaccination and decreases thereafter.

**Disclosures:**

**All Authors**: No reported disclosures

